# Obesity and endocrine-related cancer: The important role of IGF-1

**DOI:** 10.3389/fendo.2023.1093257

**Published:** 2023-01-23

**Authors:** Wentao Zhong, Xueqing Wang, Yufei Wang, Guoqian Sun, Jia Zhang, Zhuo Li

**Affiliations:** Department of Endocrinology and Metabolism, First Hospital of Jilin University, Changchun, Jilin, China

**Keywords:** obesity, endocrine-related cancer, insulin-like growth factor-1(IGF-1R), insulin like growth factor-1 receptor (IGF-1R), mechanism

## Abstract

Obesity is increasingly becoming a global epidemic of concern and is considered a risk factor for several endocrine-related cancers. Moreover, obesity is associated with cancer development and poor prognosis. As a metabolic abnormality, obesity leads to a series of changes in insulin, IGF-1, sex hormones, IGFBPs, and adipokines. Among these factors, IGF-1 plays an important role in obesity-related endocrine cancers. This review describes the role of obesity in endocrine-related cancers, such as prostate cancer, breast cancer and pancreatic cancer, focusing on the mechanism of IGF-1 and the crosstalk with estrogen and adipokines. In addition, this review briefly introduces the current status of IGF-1R inhibitors in clinical practice and shows the prospect of IGF-1R inhibitors in combination with other anticancer drugs.

## 1 Introduction

Obesity is a risk factor for several chronic diseases, such as hypertension, type 2 diabetes, dyslipidemia and cardiovascular disease. Epidemiological studies have shown that obesity is also a risk factor for certain types of cancer, such as postmenopausal breast cancer, prostate cancer, endometrial cancer, pancreatic cancer and thyroid cancer. Furthermore, a growing number of studies indicate that obesity predicts unfavorable outcomes for cancers (1). Obesity, a clinical marker of insulin resistance and metabolic syndrome, is associated with multiple biological metabolic changes, such as hyperinsulinemia, an increase in free fatty acid levels and triglycerides and hypoHDL-cholesterol. Hyperinsulinemia induces a decrease in insulin-like growth factor binding protein-1 and 2 (IGFBP-1 and 2) and reduces sex hormone binding globulin (SHBG) levels, resulting in an increase in free estrogen and androgen and insulin-like growth factor-1 (IGF-1) levels (2). The IGF system plays an important role in normal growth and development as well as in a variety of pathological situations, particularly tumorigenesis. Obesity is associated with an increased incidence of cancers arising from tissues responsive to estrogenic stimulation, including the endometrium, breast, and prostate (3). In postmenopausal women, elevated bioavailable plasma estrogen levels are related to an increased risk of breast cancer and endometrial cancer (4). Sex steroid alterations are associated with prostate cancer development and progression. This review will focus on the effects of IGF-1 on the relationships between obesity and endocrine-related cancer, especially on prostate cancer, breast cancer and pancreatic cancer.

## 2 Insulin-like growth factor-1

Insulin-like growth factors (IGFs) were first described in the late 1950s as skeletal growth factors produced in the liver in response to pituitary growth hormone (GH), and these growth factors play a fundamental role in regulating somatic growth according to nutritional conditions. IGFs, a group of polypeptide substances with growth-promoting effects, consist of IGF-1 and IGF-2. IGF-1 is produced in the liver, secreted into the circulation and acts in target tissues. In addition to the liver, IGF-1 is also produced in most extrahepatic tissues and functions as an endocrine, autocrine and paracrine growth stimulator to regulate cell growth (5). IGF-1 is a major target gene of growth hormone, and its product mediates many of the actions of growth hormone on growth and development. IGF action is also important in the development of specific organs, such as in the nervous system, in which IGF signaling regulates neuronal proliferation, apoptosis and cell survival. However, the IGF system has been implicated in various pathophysiological conditions and plays a particularly prominent role in the development and progression of human cancer (6). A growing body of epidemiological data suggests that high levels of circulating IGF-1 constitute a risk factor for the development of breast, prostate, colon, and lung cancer.

IGF-1 receptor (IGF-1R) is not mutated in most cancers and has a high degree of structural homology with insulin receptor (INSR), particularly in the tyrosine domain, which can form a heterodimer with each other and signal through many common mediators, but the two receptor signaling axes exhibit marked functional variance (7, 8). In addition, the expression levels of IGF-1R and INSR are predictive of cancer outcome. Experimentally, the modulation of IGF-1R activity affects the growth of many types of tumor cells. As a result of these findings, intensive effort is being directed toward investigating the utility of the IGF system as both a diagnostic marker and a therapeutic target in cancer therapy (6).

Six high-affinity IGF-binding proteins (IGFBP) are described at present. They are synthesized by several cell types, mostly from the fibroblast lineage, which regulate IGF-1 and IGF-2. In this family, IGFBP-3 is the most abundant IGFBP in blood, and IGFBP-2 is the second most abundant IGFBP. The IGF-1-IGFBP-3 complex and IGF1-IGFBP-5 complex bind to a third protein termed the acid labile subunit (9, 10). IGFBP-4, 5 and 6 are present in lower concentrations and appear to be less important for the regulation of free IGF concentrations in serum (5). More than 99% IGF-1 is bound to IGFBPs, which can increase the half-life of IGF to some extent, and their binding affinity for IGF is nearly 10 times higher than that for IGF-1R (10, 11). Therefore, the function and concentration of IGFBPs are critical to regulate the biological actions of IGFs.

## 3 Endocrine-related cancer

Circulating insulin and IGF-1 increase in the obese state, and sex hormones, adipokines and other inflammatory factors also vary (12). Both IGF and insulin can increase the expression of IGF-1R and INSR, which forms a functional network of interactions. In addition, IGFBPs are produced at lower levels, which reduces their inhibitory effects on insulin/IGF. IGF-1 has been studied more frequently in prostate cancer. In addition to IGF-1, estrogen is critical in breast cancer, and adiponectin is receiving increasing attention in pancreatic cancer; leptin and adiponectin have been relatively less studied in endocrine-related cancers. To varying degrees, these biological factors play a role in endocrine-related cancers.

### 3.1 Prostate cancer

A correlation between obesity and prostate cancer risk has been reported in a number of studies, especially in abdominal obesity, with a linear relationship between increasing BMI and prostate cancer (13). Moreover, obesity is associated with an increased risk of recurrence after treatment, advanced cancer progression and prostate cancer-specific mortality for prostate cancer patients (14, 15) Obesity and chronic hyperinsulinemia are known to reduce the production of IGFBPs and increase IGF-1 biological activity (16). The insulin/IGF-1 axis is associated with obesity-induced prostate carcinogenesis *via* the phosphatidylinostitol-3 kinase (PI3K)/Akt/mTOR pathways. Using Hi-Myc/HIT mice, Wang et al. showed that IGF-1 promotes prostate cancer and that the IGF-1/AKT/FOXO3A/BIM pathway plays an important role (17). Prostate cancer cells overexpress IGF-1R and INSR (18). *In vitro* and *in vivo* experiments suggest that both IGF-1R and INSR promote angiogenesis in prostate cancer (19). Sayeed et al. showed that IGF-IR signaling strictly regulates prosurvival signaling in prostate cancer by controlling the expression of a5b1 integrin, which indicates that IGF-1R and INSR promote the growth and invasion of prostate cancer (20). Therefore, changes in IGFS in obese people may increase the potential risk of prostate cancer.

According to a recent study by Markers et al., the serum levels of steroid hormones were not associated with prostate cancer risk in obese men, which is consistent with most previous studies (21). However, many studies have indicated that low testosterone and elevated estrogen levels in obese men are correlated with the development of prostate cancer (22). To clarify these contradictory results, further investigation of the underlying mechanisms is still needed. Leptin has prostate cancer-promoting effects and is positively correlated with fat mass, while adiponectin has anticancer effects and is negatively correlated with BMI (23, 24). The ratio between leptin and adiponectin is imbalanced in obese individuals, leading to abnormalities in the AMPK and mTOR signaling pathways, which may influence prostate cancer development (25). In addition, adipose tissue itself is also associated with prostate cancer. Periprostatic adipose tissue secretes a variety of inflammatory factors and creates a tumor microenvironment that promotes the development of prostate cancer (26–29).

### 3.2 Breast cancer

Obesity is associated with the occurrence and progression of breast cancer, especially postmenopausal ER+/PR+ breast cancer (30, 31). According to a systematic evaluation, higher levels of IGF-1, IGFBP-3 and leptin and lower levels of adiponectin among obesity-associated protein biomarkers are correlated with an increased breast cancer risk (32). The association between IGFBP-3 and breast cancer risk is reportedly due to its interaction with IGF-1, and this interaction can be eliminated by the adjustment of IGF-1, suggesting that IGF-1 itself is an inducing factor of breast cancer (33).

In addition to the direct regulation of estrogen and these obesity-associated protein biomarkers on breast cancer cells, these biological factors exhibit crosstalk. The role of IGF-1 is mediated by IGF-1R, which is overexpressed in breast cancer (34, 35). IGF-1R promotes breast cancer by altering the expression of proliferation and survival genes through the Ras/Raf/MAPK and PI3K/Akt signaling pathways (36, 37). Insulin can also promote breast cancer *via* these signaling pathways (38). The estrogen-induced production of ROS leads to DNA damage, while estrogen itself inhibits the DNA damage response and can promote cell proliferation, which is the background of estrogen-induced breast cancer (39–41). Postmenopausal adipose tissue is an important site of estrogen production. The rate of estrogen conversion is higher in obese postmenopausal women, and obesity increases estrogen levels in ER receptor-positive breast cancer tissue, which increases the risk associated with breast cancer (42, 43). This risk is further increased by a reduction in SHBG in the liver of individuals with obesity and an increase in the concentration of non-SHBG-bound estradiol (E2, the most abundant and active estrogen) (44, 45). Similar to IGF-1, estrogen can also play a role in breast cancer through the MAPK and PI3K/Akt signaling pathways, and estrogen and IGF-1 exhibit crosstalk. E2 accelerates and enhances the binding of ERα to IGF-1R, and ERα, activated by the Ras-MAPK cascade of the growth factor signaling pathway, induces the phosphorylation of Akt and ERK1/2 after rapid binding to IGF-1R, thereby enlarging IGF-1R signaling (46). Leptin, which is associated with estrogen, also contributes to breast cancer. It may upregulate the PI3K/Akt pathway, MAPK pathway and STAT3 pathway, the downstream signaling pathways of Ob-R, which stimulate oncogenesis (47). Interestingly, elevated cAMP may inhibit leptin-induced migration of highly aggressive breast cancer cells MDA-MB-231 by suppressing ERK1/2 and STAT3 signaling pathway (48, 49). Leptin also promotes the proliferation of breast cancer cells by activating the Wnt/β-catenin pathway (50). Aromatase is the rate-limiting enzyme for estrogen synthesis, and leptin can induce aromatase expression through COX-2 expression in breast cancer cells and increase estrogen synthesis, promoting the progression of breast cancer (51, 52). Zahid et al. showed that the increase in aromatase expression in the obese state may be mediated by leptin *via* the P53-HIF1α/PKM2-aromatase axis (53). In addition, Morad et al. suggested that estrogen can in turn increase the expression of leptin and leptin receptors (54). In ERα-negative breast cancer, adiponectin inhibited IGF-1-induced cell migration, whereas in ERα-positive breast cancer, low concentrations of adiponectin promoted IGF-1R phosphorylation and thus enhanced IGF-1/IGF-1R signaling (55, 56). The concentration of adiponectin tends to decrease in obese states, which may reduce the protective effect and increase the risk of breast cancer. In conclusion, in obese women, especially postmenopausal women, the role of estrogen and obesity-associated protein biomarkers in breast cancer is complicated due to their own changes and crosstalk.

### 3.3 Pancreatic cancer

Epidemiological surveys show that obesity is associated with pancreatic cancer morbidity and mortality, and the morbidity of pancreatic cancer increases with increasing BMI (57, 58). Since approximately 90% of pancreatic cancers are pancreatic ductal carcinoma (PDAC), many models have been built with PDAC. Various events are considered to elevate the risk of pancreatic cancer in obesity, such as the increase in IGF-1. A survey including 105 patients showed that IGF-1R was overexpressed in more than half of the PDAC samples (59). Du et al. indicated that high IGF-1R expression was associated with shorter overall survival and relapse in patients (60). In PDAC, both insulin and IGF-1 are considered to play a role in cancer development and progression, with the PI3K and MAPK signaling pathways as its central pathways, and these signals are enhanced in obesity (61, 62). Tian et al. inhibited the PI3K/Akt signaling pathway by knocking down the IGF-1R gene, thereby inhibiting the proliferation of pancreatic cancer cells and increasing the sensitivity of anticancer drugs, which to some extent proves the role of IGF-1 in pancreatic cancer (63). Oncogenic KRAS mutation occurs in approximately 90% of PDAC, and MEK/ERK is one of the main effector pathways of KRAS signaling (64). MEK/ERK-stimulated IGF-1R signaling is required for murine pancreatic epithelial cell transformation, which suggests a critical role for IGF-1R signaling in pancreatic carcinogenesis (65). In addition, leptin, adiponectin and inflammatory factors also play an important role in the association between obesity and pancreatic cancer (66, 67). Adiponectin acts by binding to AdipoRs to activate downstream signaling pathways and AdipoRon is a synthetic small molecule AdipoR agonist that binds to the AdipoR1 and AdipoR2. AdipoRon induces pancreatic cancer cell death by activating ERK1/2, however, obesity weakens this anti-cancer effect (68, 69). Messaggio et al. demonstrated that leptin promotes pancreatic tumor growth through altered pathways of STAT3 and PI3K/AKT signaling, whereas AdipoRon inhibits leptin-induced STAT3 activation and pancreatic tumor growth *in vivo (* 70, 71). Recently, Ragone et al. combined gemcitabine, a first-line agent for pancreatic cancer, with AdipoRon to enhance growth inhibition in human PDAC cell lines, which may be associated with the AdipoRon mediated p44/42 MAPK(ERK1/2) pathway (72).

### 3.4 Other endocrine-related cancers

Ovarian, endometrial and thyroid cancers are also associated with obesity. In ovarian cancer, epidemiological studies show that obesity may promote the peritoneal dissemination of ovarian cancer and increase patient mortality (73, 74). Ignacio et al. suggest that obesity may accelerate the peritoneal dissemination of ovarian cancer by producing more proinflammatory chemokines and recruiting macrophages (75). Leptin helps maintain the cancer stem cell-like properties of ovarian cancer cells and stimulates the migration and invasion of ovarian cancer cells (76). IGF-IR mediates TGFβ for the maintenance of EMT transformation, angiogenesis and extracellular matrix remodeling (77). Together, these factors contribute to the development of ovarian cancer. In endometrial cancer, epidemiological studies have shown that obesity is associated with an increased incidence of endometrial cancer and is correlated with recurrence and death in patients with endometrial cancer (78). Merritt et al. indicated that estrogen, insulin, and IGF-1 are associated with the multistage process of endometrial carcinogenesis (79). Estrogen can stimulate cell proliferation by increasing IGF-1 synthesis and IGF-IR expression in the uterus *via* ERα (80). In addition, in the absence of IGFBP-1 synthesis in obesity, IGF-1 may further induce the abnormal proliferation of endometrial cells, which is a possible pathway for endometrial cancer formation (81). In thyroid cancer, a cohort study collected more than 450,000 samples from 1995 to 2015 and suggested that being overweight and obesity may be important causes of the increased incidence of thyroid cancer (82). IGF-1 may play a role in the association between obesity and thyroid cancer (83). IGF-1 is positively associated with the risk of developing thyroid cancer (84). Yang et al. suggested that IGF-1 promotes the proliferation and invasion of thyroid-like carcinoma through the STAT3 pathway (85). Studies have also indicated a role for the IGF axis in thyroid tumorigenesis (86).

## 4 Therapies targeting the IGF family

In the face of increasing cancer incidence, researchers have targeted the IGF axis for therapeutic intervention due to its role in cancer.Three main approaches are used to target IGFs, including IGF-1R monoclonal antibodies, IGF-1R tyrosine kinase inhibitors (TKIs) and IGF-1/-2 blocking monoclonal antibodies. IGF-1R monoclonal antibodies (IGF-1R mAb, such as cixutumumab, figitumumab and ganitumab) function mainly by blocking ligand-receptor action, inducing the internalization/degradation of IGF-1R and partially downregulating IGF-1R/INSR hybrid receptors (87, 88). Among IGF-1R TKIs, ATP-competitive TKIs act by competing for the binding site of the IGF-1R kinase domain to ATP, which inhibits both IGF-1R and INSR. However, non-ATP competitive IGF-1R inhibitors do not influence INSR (89, 90). IGF-1/-2 blocking mAb blocks IGF-1R and INSR-A and their hybrid receptors from transducing proliferative/anti-apoptotic signals by binding IGF-1 and IGF-2 without affecting INSR-B and insulin function (91). For more than a decade, researchers have conducted numerous clinical trials on these three classes of inhibitors due to the role of IGF-1 in tumor development (**Table 1**). Unfortunately, the results of most clinical trials targeting single-agent activity were disappointing. Some IGF-1R inhibitors have been discontinued or abandoned from development; for example, Pfizer decided to discontinue figitumumab (CP-751871) in 2010, and Roche shifted to teprotumumab to treat ophthalmic disease.

**Table 1:** Clinical trials of IGF-1 inhibitors as single agents.

Cancer type	Drug	Drug Type	Clinical trial Phase	Number of patiens	Response	Trial ID	Ref.
Postmenopausal women with hormone receptor–positive breast cancer	Cixutumumab	Anti-IGF-1R mAb	II	n=93(31 in the cixutumumab alone group)	No significant clinical effect observed with cixutumumab alone	NCT00728949	(92)
Advanced hepatocellular carcinoma	Cixutumumab	Anti-IGF-1R mAb	II	Qnly stage 1 was accrued: n= 24	No significant clinical effect	NCT00639509	(93)
Refractory Solid Tumors	Cixutumumab	Anti-IGF-1R mAb	II	n=116	Limited objective single-agent activity of cixutumumab was observed; PR:20%(neuroblastoma with only MIBG evaluable disease ), SD:15%(patients with a variety of solid tumor types)	NCT00831844	(94)
Recurrent or refractory TETs	Cixutumumab	Anti-IGF-1R mAb	II	n=49(37 thymomas;12 thymic carcinomas)	PR :14%,SD :76%(thymoma cohort); PR:0,SD:42%(thymic carcinoma cohort)	NCT00965250	(95)
Rhabdomyosar-coma; Leiomyosarcoma; Adipocytic sarcoma; Synovial sarcoma; Ewing family of tumours (including ES-1 and peripheral neuroectodermal tumour)	Cixutumumab	Anti-IGF-1R mAb	II	n=113(all tiers except adipocytic sarcoma were closed after stage 1 due to futility)	PFR:12%(rhabdomyosarcoma, n = 17), 14%( leiomyosarcoma, n=22), 32%(adipocytic sarcoma, n=37), 18%(synovial sarcoma, n=17), 11%(Ewing family of tumours, n=18)	NCT00668148	(96)
Ewing sarcoma, osteosarcoma and other sarcomas	Figitumumab	Anti-IGF-1R mAb	I/II	Phase Iportion n=31, phase II portion n=107	phaseIIportion ORR=14.2%	NCT00560235	(97)
Neuroendocrine tumor	MK-0646	Anti-IGF-1R mAb	II	n=25	inactive as a single agent	NCT00610129	(98)
Previously treated, locally advanced or metastatic NSCLC of the SCC or AC subtypes	AXL1717	IGF-1R TKI	II	n=99(58 in the AXL1717 group)	12-week PFS: 25.9%(AXL1717 group), 39.0%(docetaxel group),without any statistically significant differences	NCT01561456	(99)
Adrenocortical carcinoma	Linsitinib	IGF-1R/INSR TKI	III	n=135	No difference in overall survival was noted between linsitinib and placebo	NCT00924989	(100)
Metastatic castrate resistant prostate cancer	Linsitinib	IGF-1R/INSR TKI	II	n=18	No significant PSA or objective response; without any effect on circulating tumor cells or survival benefit	NCT01533246	(101)
advanced/metastatic solid cancers	Xentuzumab	IGF-1/IGF-2-neutralizing Ab	I	Study 1280.1:n=61; Study 1280.2:n=64	Preliminary anti-tumour activity; Study 1280.1 part 1:PR(n=2),SD(n=3); Study 1280.1 part2: SD(n=3); Study 1280.2: no objective responses; SD(n=2 in part1)	NCT01403974; NCT01317420	(102)
advanced solid tumors	xentuzumab	IGF-1/IGF-2-neutralizing Ab	I	n=21	Preliminary anti-tumour activity; ORR=9.5% SD=19.0%	NCT02145741	(103)

IGF-1R, insulin-like growth factor receptor 1; INSR, insulin receptor; PR, partial response; SD, stable disease; ORR, objective response rate; AE, most common grade 3-4 adverse events; MIBG, meta-iodo-benzyl-guanidine; PFR, progression-free survival rate; TETs, thymic epithelial tumors; TKI, tyrosine kinase inhibitor; NSCLC, non-small cell lung cancer; SCC, squamous cell carcinoma; AC, adenocarcinoma; PFS, progression-free survival.

One possible reason for the resistance of cancer cells to IGF-1R inhibitors is the compensatory activation of RTK signaling; therefore, several combination therapies have recently emerged to improve efficacy (104, 105), such as IGF-1R inhibitors in combination with other RTK inhibitors. EGFR is an important factor in the RTK signaling pathway, and the IGF-1/2 neutralizing antibody m708.5 exhibits a strong synergistic effect with gefitinib, which shows benefit in the treatment of neuroblastoma and breast cancer (106). IGF-1R inhibitors are valuable in endocrine-related tumors. For example, the activation of the IGF-1R pathway is associated with tamoxifen resistance, while the elimination of IGF-1R signaling with linsitinib could restore the sensitivity of endocrine therapy (107). Combined IGF-1R/mTOR inhibition also shows synergistic effects. In ACC, *in vitro* assays showed stronger antiproliferative activity in combination with sirolimus or everolimus than with linsitinib (108). A phase I clinical trial of ridaforolimus in combination with anti-IGF1R mAb dalozumab also demonstrated clinical activity in advanced cancer (109). Some experimental support also exists for the combination of IGF-1R inhibitors with therapeutic approaches to induce DNA damage. Most invasive breast cancers have insulin/IGF-1R signaling activation, and xentuzumab in combination with paclitaxel can effectively reduce metastasis incidence and metastatic burden in preclinical mouse models (110). IGF-1R is also involved in radiotherapy resistance. Low IGF-1R expression was demonstrated to increase the sensitivity of CRC to radiation therapy when treated with NVP-ADW742 in CRC cells (111, 112). Some anticancer drugs containing IGF-1R inhibitors have entered in phase III clinical trials, but clinical benefit is not significant. More clinical trials are needed to prove the effectiveness of the combination therapy.

## 5 Conclusion

**Figure1:**
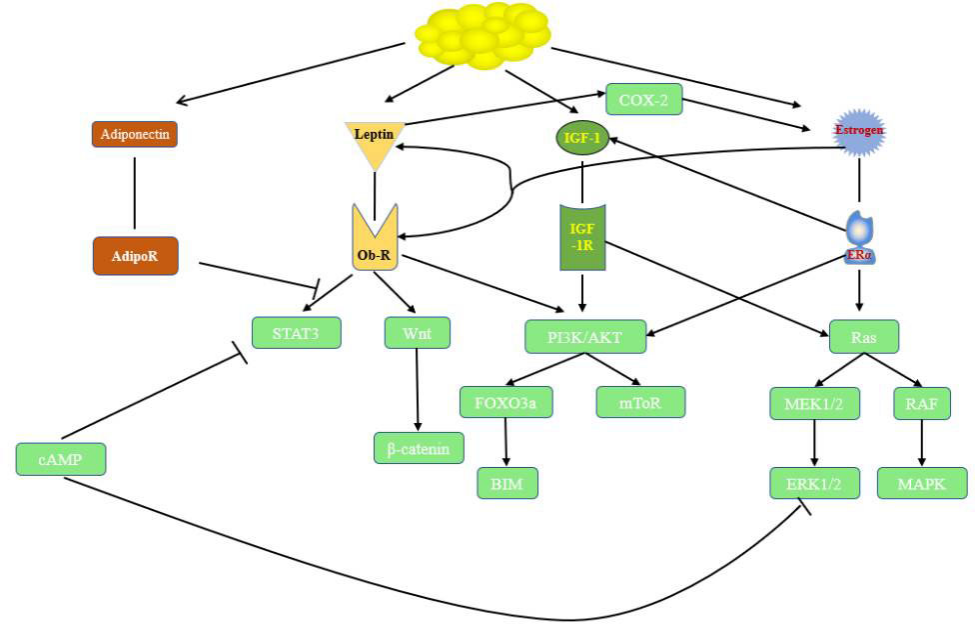
Relationships and roles in endocrine-related cancer. cAMP, cyclic adenosine monophosphate; ObR, leptin receptor; STAT3, signal transducer and activator of transcription 3; FOXO3a, Forkhead box O3; IGF-1, insulin-like growth factor; IGF-1R, insulin-like growth factor receptor 1; P13K/Akt, phosphatidylinositol 3-kinase/protein kinase B; ERα, estrogen receptor α; MAPK,mitogen-activated protein kinase;MEK,mitogen-activated protein kinase;ERK, extractor signal regulated kinase.

Obesity has been called the disease of modern civilization and is a heavy disease burden for society. Disorders of insulin, IGF-1, sex hormones, adipokines and inflammatory factors have been observed in numerous studies in the presence of obesity. Obesity is also recognized as a factor associated with cancer, and adipose tissue, which is an active endocrine organ, strengthens the link between obesity and endocrine-related cancers. IGF-1 plays an important role in obesity-associated endocrine-related cancers, promoting cancer development mainly through the PI3K/AKT and MAPK pathways. Insulin, IGF-1, sex hormones and adipokines also exhibit crosstalk to synergistically function in this process. The relationships among them and their roles are shown in **Figure 1**. Investigators have conducted a series of clinical trials targeting IGFs; however, most of them failed to demonstrate single-agent efficacy. Although drug combinations perform better in preclinical studies, fewer patients benefit from clinical trials. The following issues remain in the study of obesity and endocrine-related cancers: 1. the mechanism of action of obesity-related biologic factors in cancer and the crosstalk among them still need to be studied further; 2. whether obesity-related biologic factors can be used as biomarkers to evaluate endocrine-related cancers has not been fully investigated; 3. suitable predictive biomarkers urgently need to be identified in clinical trials to design reasonable joint therapeutic strategies accordingly. If these problems are solved in the future, IGF inhibitors can play a larger role in clinical practice and provide more treatment options for cancer patients.

## Author contributions

WZ wrote and revised the manuscript. ZL wrote the outline and critically revised the manuscript. WZ, XW, YW, GS and JZ contributed to literature search. WZ and XW prepared the table and figure. All authors have read and given approval of the final manuscript.
